# Supplementary material for: Using Sensors for Player Development: Assessing Biomechanical Factors Related to Pitch Command and Velocity
Source: Sensors (Basel). 2022 Nov 4;22(21):8488. doi: 10.3390/s22218488 (PMC9655623; doi:10.3390/s22218488)
Supplement: Supplementary file 1 [file sensors-22-08488-s001.zip › sensors-1969593-supplementary.pdf]

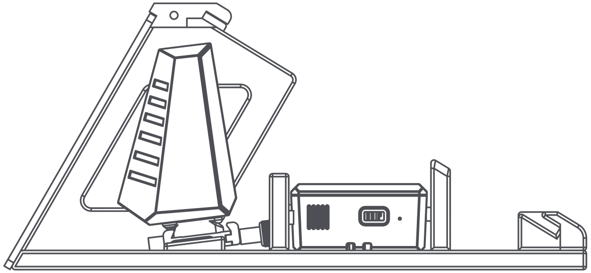

Rapsodo®

PITCHING 2.0

USER MANUAL

Powered by: Rapsodo

CONTENTS

What's Inside the Box 1

Cloud + App 2

Overview 3

Operating the Monitor 4

Understanding the Monitor LED Displays 5

Operating the Rapsodo Computing Engine (RCE) 6

Understanding the RCE LED Displays 7

Charging the Devices 8

Pairing the RCE and the App 9

Device Placement and Setup 10

Calibration 11

Troubleshooting Table 12

Specifications Ball Flight 13

Specifications Release 14

General Information 15

Support / Contact 16

Rapsodo®

WHAT'S INSIDE THE BOX

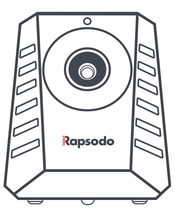

Monitor

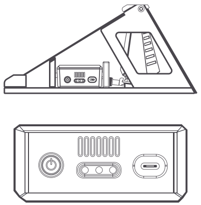

Protective Tank & RCE

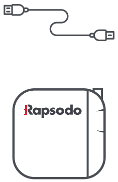

USB Cables & Charger

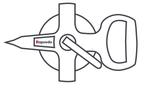

Measuring Tape

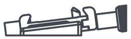

Monitor Clip

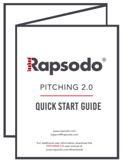

Quick Start Guide

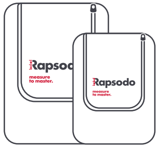

Protective Cases

CLOUD + APP

1. Create a coach account and player profiles at **cloud.rapsodo.com**

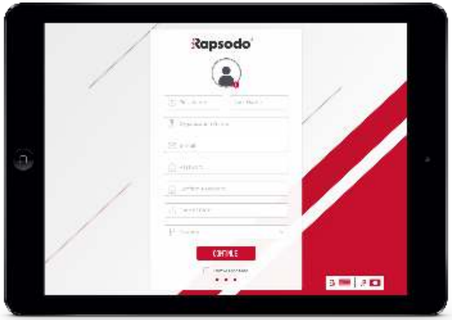

2. This will be your username and password to login to both the cloud and applications

3. The Rapsodo Pitching App must be downloaded in order to use **PITCHING 2.0**

PITCHING 2.0

4. To download go to the **Apple App Store** and search **Rapsodo PITCHING**

Available on the App Store

App Requirements:

- iOS 10.3 or later
- iPad Air 2 or newer

OVERVIEW

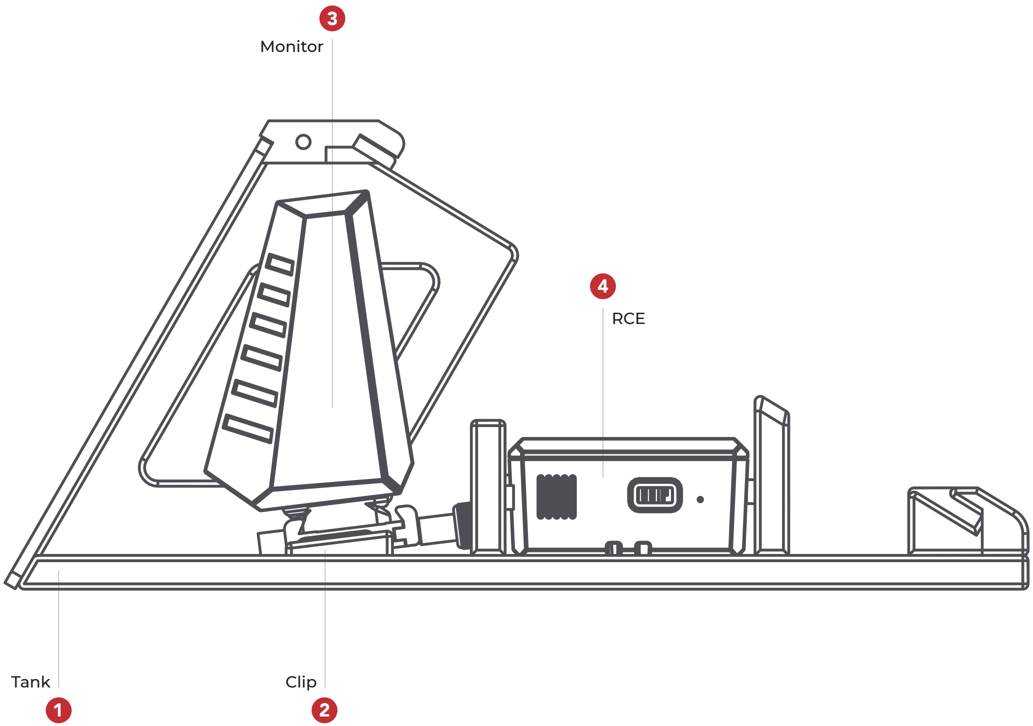

OPERATING THE MONITOR

Turning the Monitor ON

- To turn the monitor on, press the power button
- The front LED display will turn RED indicating the monitor is on but not ready to measure
- The rear LED will turn RED when ready to connect

Turning the Monitor OFF

- Press the power button

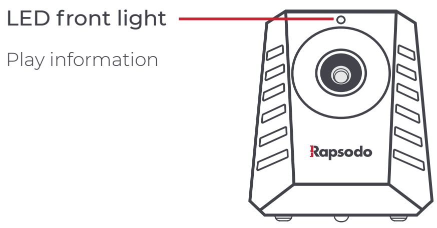

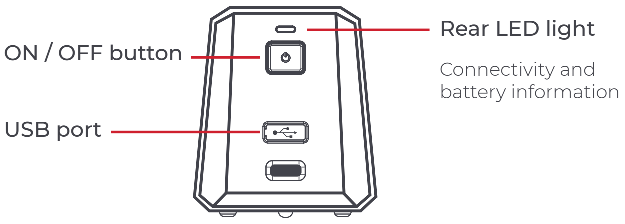

UNDERSTANDING THE MONITOR LED DISPLAYS

Front LED Display

The front LED display provides information on the monitor status, and will give the "**green light**" to pitch when the device is ready to measure.

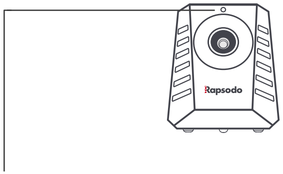

RED LIGHT "NOT READY"

Monitor is switched on, but not ready to measure.

GREEN LIGHT "PITCH"

Monitor is ready for pitching. When appearing after blue light just after a pitch: data is ready to be viewed on app, and ready for next pitch.

BLUE LIGHT "PROCESSING"

Monitor is processing data after a valid pitch. Wait for the green light to appear which should take only a few seconds.

Rear LED Display

The rear LED display provides information on connectivity and battery. This display will be especially important when pairing the app with the monitor.

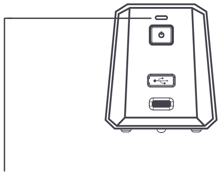

RED LIGHT

Not connected to RCE, waiting for USB sync.

GREEN LIGHT

Successfully connected to RCE through USB.

RED FLASHING LIGHT

Warning for low battery, please recharge monitor.

AMBER LIGHT

Switched off, and charging.

OFF

If USB is plugged into a charging device or PC, it means the battery is fully charged.

Rapsodo®

OPERATING THE RAPSODO COMPUTING ENGINE (RCE)

Turning the RCE ON

- To turn the RCE on, press the power button
- After 10-15 seconds the power LED will turn on and the RCE will be ready to connect to the monitor

Turning the RCE OFF

- Press the power button
- It will take 10-15 seconds for the RCE to turn off

FRONT

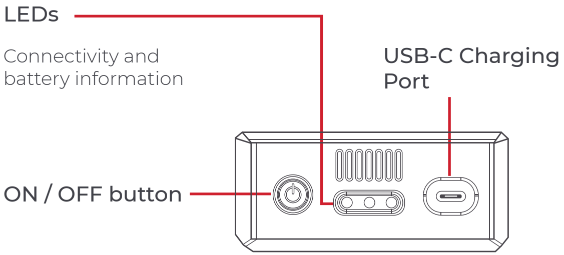

BACK

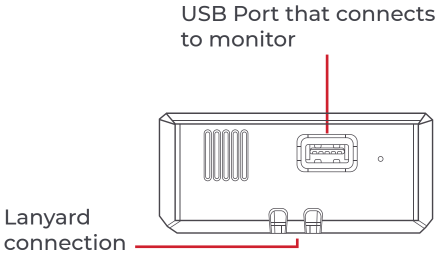

UNDERSTANDING THE RCE LED DISPLAYS

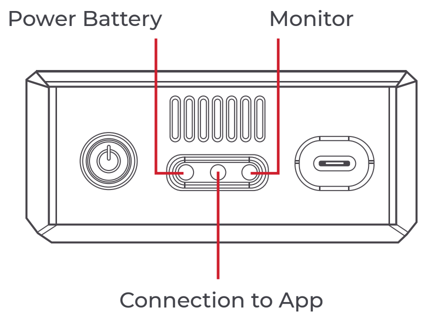

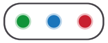 RCE and monitor connected

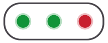 RCE and monitor connected to application, needs calibrated or on ‘Pause Mode’

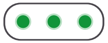 Ready to record data

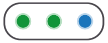 Data processing

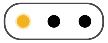 RCE switched off and charging

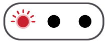 Warning for low battery, please recharge monitor

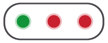 RCE turned on, not connected to app and monitor

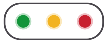 RCE turned on, connected to app but not the monitor

CHARGING THE DEVICES

Charging the Monitor

- The battery can be charged by connecting the included USB cable into the port on the rear monitor panel
- The rear LED will show AMBER indicating the monitor is charging
- The rear LED will switch off indicating the device has been sufficiently charged
- The LED indication will blink red indicating the battery charge is getting low and the battery should be charged

Charging the RCE

- The battery can be charged by connecting the included USB-C cable to the USB-C charging port (located on the side of the power button)
- The power LED will show AMBER indicating the monitor is charging
- The power LED will switch off indicating the device has been sufficiently charged
- The LED indication will blink red indicating the battery charge is getting low and the battery should be charged

The monitor and RCE must be charged separately to ensure a complete charge for each device

PAIRING THE RCE AND THE APP

1. Connect the RCE to the monitor via USB to mini USB

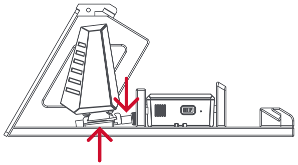

2. Press the power button on the monitor and RCE

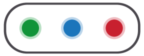

3. Connect to Rapsodo Wifi in iPad settings

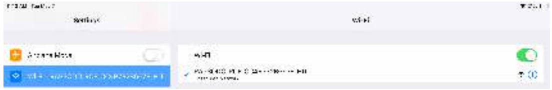

4. Open the Rapsodo PITCHING 2.0 app and press the Wifi connection button

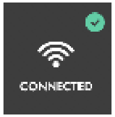

Rapsodo®

DEVICE PLACEMENT AND SETUP

Rapsodo PITCHING 2.0 operates in a tank. Correct positioning is important to get accurate results.

1. Internal Tank Set-Up

1. Attach monitor to mount plate
2. Place RCE in housing
3. Connect USB from monitor to RCE

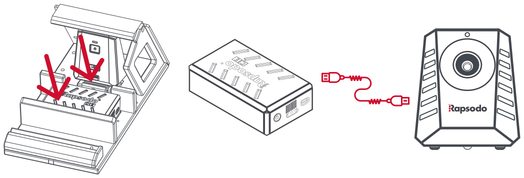

2. Tank Positioning

Rapsodo PITCHING 2.0 should be placed 15’6” from the front of home plate and the camera opening should face the pitcher. The tank should be placed on an even surface and SHOULD NOT be used in the rain or wet conditions.

⚠ Rapsodo PITCHING 2.0 should only be used at the designated distance and on a flat surface. Improper placement may cause injury.

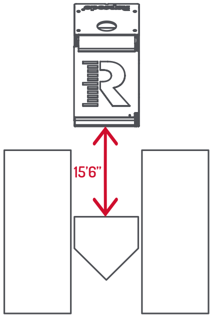

3. Calibration

1. Align monitor angles with help of the application
2.Use azimuth view and tape measure to position the tank directly in the center of the pitching lane

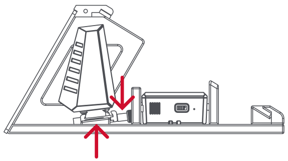

CALIBRATION

1. Press calibration icon inside app

2. Adjust monitor angles to
   0 degrees and 14 degrees

3. To calibrate the monitor have a person stand on the center of the pitching rubber. The red line in the azimuth view should go directly down the middle of the pitching rubber.

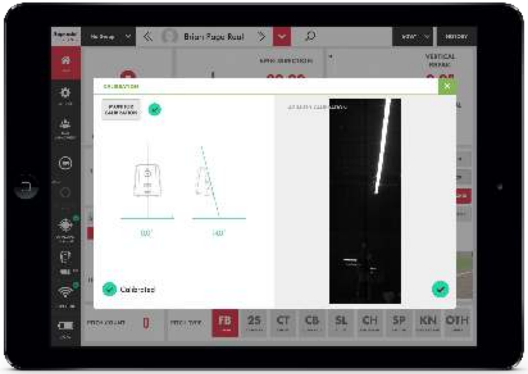

Rapsodo®

TROUBLESHOOTING TABLE

**App shows low monitor battery**	Charge RCE and monitor separately
**Results seem inaccurate**	Make sure the tank is placed at 15’6” and you have correctly calibrated the monitor
**Can’t connect to app**	Connect to wifi and calibrate from the play tab inside the app
**Data not syncing to cloud**	Disconnect from RCE wifi and connect to an internet based wifi. Press sync and leave app open

SPECIFICATIONS BALL FLIGHT

Velocity
How fast a pitch is traveling during flight, measured in miles per hour (MPH).

Spin Rate
The rate at which the ball spins during flight, measured in rotations per minute (RPM). There is a stronger correlation between spin rate and swinging-strike percentage than between velocity and swinging-strike percentage.

True Spin Rate
The spin directly impacting the movement of a pitch. Also known as “useful spin,” it is perpendicular to the direction the ball is traveling, deflecting the otherwise straight horizontal and vertical path of the ball.

Spin Direction
The tilt or angle of the baseball from the release point, measured in degrees, created by the Magnus effect. The Magnus Effect is created by the air pressure surrounding the spinning baseball on its path to home plate.

Strike Zone Analysis
Instantly see Strike Zone feedback while automatically tracking Strike Percentage and Heatmap Breakdowns.

Spin Efficiency
The percentage of spin directly impacting the movement of a pitch. Spin Efficiency is the ratio of true spin to total spin.

Pitch Break
How much a ball has moved when it crosses the strike zone compared to what its position would have been without spin. Break is measured horizontally and vertically in inches.

3D Trajectory
The ballflight is show in two trajectories. A dotted line represents what the pitch would have done without spin and the solid line represents the actual movement of the pitch.

SPECIFICATIONS RELEASE

Release Height
The height of the ball at the point of release.

Release Angle
The vertical angle at which the ball was released out of the hand.

Release Side
The distance away from the center of the rubber from the left (-) or the right (+)

Horizontal Angle
The horizontal angle the ball was released out of the hand from the left (-) or the right (+)

Release Extension
The distance away from the front of the rubber the ball was released.

GENERAL INFORMATION

Interference
Rapsodo PITCHING 2.0 is an extremely sensitive and accurate data-tracking monitor. While Rapsodo PITCHING 2.0 hardware has been engineered to achieve accuracy within a regular baseball environment; many everyday items like electrical appliances, computers, cell phones, bulbs, etc. may produce electrical or optical interference that can generate false readings. You need to be aware of these kinds of products in the environment where Rapsodo PITCHING 2.0 is used, as they may cause interferences.

Use In Well-Lit Environments
Rapsodo PITCHING 2.0 should be used in well lit locations. Performance may vary in certain lighting conditions, particularly in low light conditions, which may result in incorrect measurements.

Do not use Rapsodo Pitching 2.0 in Wet Conditions
Rapsodo PITCHING 2.0 is not water resistant. Do NOT expose the device to water as it may get damaged.

App Updates
Rapsodo will periodically update and improve the features of the Rapsodo Baseball app. It is recommended that users regularly download the latest version to make for the highest monitor performance.

Return Policy and Warranty
Rapsodo provides a (1) one-year limited warranty for manufacturing defects. Under conditions of the warranty agreement, purchasers will be entitled to repair or replacement for defective Rapsodo products. This warranty begins from the date of initial purchase. Rapsodo may also choose to refund the purchase price of the product to the purchaser, at its sole discretion. Warranty detailed information and return policy can be found online at www.rapsodo.com/policy-warranty.

Regulatory notice: Waste electrical and electronic equipment
The symbol on the product or its packaging signifies that this product has to be disposed separately from ordinary household wastes at its end of life. Please kindly be aware that this is your responsibility to dispose electronic equipment at recycling centres in order to help conserve natural resources. Each country in the European Union should have its collection centres for electrical and electronic equipment recycling. For information about your recycling drop off point, please contact your local electrical and electronic equipment waste management authority. Do not dispose of the Rapsodo device with household waste. Batteries are not to be disposed of in municipal waste stream and require separate collection. Disposal of the packaging and your Rapsodo should be done in accordance with local regulations.

SUPPORT / CONTACT

If you have any questions or need any assistance, please contact us at:

Email: support@rapsodo.com

Phone: 1.844.772.7763

www.rapsodo.com
